# Prevalence of Dental Anomalies in the Patient with Cleft Lip and Palate Visiting a Tertiary Care Hospital

**DOI:** 10.31729/jnma.5149

**Published:** 2020-08-31

**Authors:** Leeza Pradhan, Pramila Shakya, Swosti Thapa, Kiran Kishor Nakarmi, Anjana Maharjan, Reshu Agrawal Sagtani, Shankar Man Rai

**Affiliations:** 1 Department of Burns, Plastic and Reconstructive Surgery, Nepal Cleft and Burn Centre, Kirtipur Hospital, Kathmandu, Nepal; 2 Department of Dentistry, Patan Academy of Health Sciences, Lalitpur, Nepal; 3 School of Public Health, Patan Academy of Health Sciences, Lalitpur, Nepal

**Keywords:** *cleft lip and palate*, *dental anomalies*, *panoramic radiograph*

## Abstract

**Introduction:**

Dental anomaly is one of the major problems in a child born with cleft lip and palate. These anomalies have deleterious effects on the dentition leading to aesthetic problems, impairment of mastication andimproper phonation. The aim of our study was to find out the prevalence of dental anomalies in patient with cleft lip and/or palate radiographically.

**Methods:**

A descriptive cross-sectional study was conducted from the 208 radiographs, collected by the convenience samplingtechnique with cleft lip and/or palate in Department of Burns, Plastic and Reconstructive Surgery, Nepal Cleft and Burn Centre, Kirtipur Hospital from January 2017 to July 2019. Ethical clearance for the study was obtained from Institutional Review Committee. Demographic data were collected and radiographs were evaluated for possible dental anomalies. Data obtained were entered and analysed in Statistical Package for Social Sciences version 23.

**Results:**

Dental anomalies were highly prevalent among cleft lip and palate patients with at least one anomaly present in 188 (90.4%) of patients with male 120 (57.4%) presenting more anomalies than female 88 (42.6%) population. The most common anomaly was dental agenesis 161 (77.9%). The prevalence of positional anomaly, morphological anomaly and supernumerary teeth were found to be 54 (26%), 33 (15.9%) and 20 (10%) respectively. Lateral incisor showed the highest incidence of agenesis among all other missing teeth 223 (65.2%).

**Conclusions:**

The prevalence of dental anomalies among patients with cleft lip and/or palate was found to be high. Tooth agenesis was the most common anomaly observed in the study with lateral incisor having the highest incidence of agenesis.

## INTRODUCTION

Cleft lip and/or palate (CL/P) constitutes to a large fraction of all birth defects.^1^ Dental anomaly is one of the major problems in children with CL/P. These anomalies may be attributed to severity of cleft or early surgical corrections^2^ and possesses problems with appearance, mastication and improper phonation.^3^

Association between dental anomalies in CL/P may be attributed to a close embryological relationship in timing and anatomical position of formation of tooth germs and the occurrence of cleft.^4^ The absence of fusion between the maxillary and medial nasal processes that results in theCL/P explains various anomalies affecting lateral incisor and the presence of supernumerary teeth.^5^

The aim of our study was to find out the prevalence of dental anomalies in the permanent dentition of individuals with cleft lip and palate.

## METHODS

A descriptive cross-sectional study was carried out in the Department of Burns, Plastic and Reconstructive Surgery at Nepal Cleft and Burn Centre, Kirtipur Hospital. Orthopantomogram (OPG) radiographs of all the patients who were previously operated for cleft lip and palate and came with residual alveolar cleft or for orthodontic treatment were included in the study. However, OPG radiographs of syndromic patient were excluded. Ethical clearance for the study was obtained from Institutional Review Commitee of Public Health Concern Trust (phect)- Nepal under which Kirtipur Hospital operates.

The sample size of 208 was calculated based on the cleft lip and palate patient visiting Kirtipur hospital i.e. 450 per year.

Sample size was calculated using the formula with finite population correction:

$$\begin{array}{ll} \text{n} & \text{= }{\text{Z}}^{\text{2}}\times \text{(p}\times \text{q)}/e^{\text{2}}\\ & \text{= }{(\text{1.96)}}^{\text{2}}\times \text{0.05}\times (1-0.5)/\text{0}{\text{.05}}^{\text{2}}\\ & \text{= }384 \end{array}$$

Where,

n = required sample size
p = prevalence of anomalies in cleft patient (50%)
q = 1-p
e = margin of error, 5%
Z = 1.96 at 95 % CI

In the Kirtipur hospital, number of cleft lip and palate patients operated/year (N): 450

Corrected sample size= n/1 + n - 1/N = 207

Therefore, the calculated sample size was 207.

Convenience sampling technique was utilized for collecting data. A total of 208 patients previously operated for cleft lip and palate patients were included in the study.

Completed clinical records from January 2017 to July 2019 were assessed to achieve the patient information like name, age, sex, type and laterality of cleft. Panoramic radiographs of these patients were examined and information of dental anomalies like missing teeth, morphological anomaly in shapes, presence of supernumerary teeth and positional anomalies like ectopic eruption, mal-aligned teeth were recorded. All the Panoramic radiographs were obtained from a single imaging centre for standardisation. The panoramic radiographs were analyzed by a single investigator who followed a systematic analysis of all the erupted and unerupted tooth number, morphological anomalies, positional anomalies and presence of supernumerary teeth in each quadrant. The evaluation of digital panoramic radiographs was carried out on a computer screen.

The data were collected and entered in MS Excel 2007 and analyzed using the Statistical Package for Social Sciences (SPSS) Version 23 software.

## RESULTS

**Figure 1.**
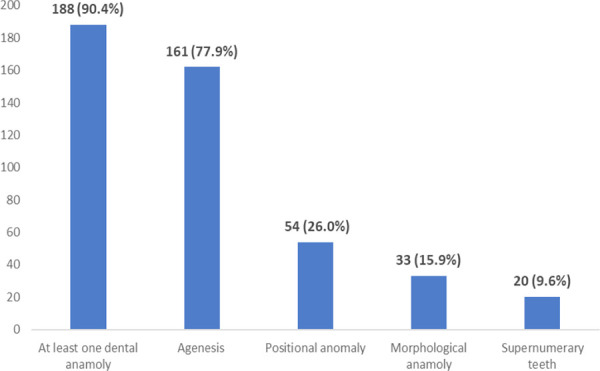
Prevalence of dental anomalies among cleft patients. (n = 208).

This study analyzed panoramic radiographs of 208 patients with cleft lip and palate. Majority 120 (57.7%) of the patients were male. The age of the patients ranged from 7 years to 27 years with average age being 12.5 years. Majority 184 (88.5%) of the study patients showed complete cleft lip and palate. Also 151 (72.7%) had unilateral clefts and 56 (26.9%) had bilateral clefts. Dental anomalies were highly prevalent among cleft lip and palate patients with at least one anomaly present in 188 (90.4%) of patients (Fig 1).

The most common dental anomaly reported among the cleft patients of the study was agenesis which was seen among more than 3/4^th^ i.e.77.9% of the patients. Maxillary laterals were the most common 223 (65.2%) teeth to be missing in cleft populations with higher prevalence 126 (56.5%)on left side compared to 97 (43.49%) on right side.

The prevalence of positional anomalies like ectopic eruption and malalignment was 54 (26 %) while morphological anomalies like peg shaped laterals was seen among 33 (15.9%) of the patients. The supernumerary teeth were seen among 20 (10%) of the patients. Thus, presence of at least one of the dental anomalies was reported in more than 90 % of cleft patients of this study.

Among morphological anomalies peg shaped maxillary lateral incisors were the most common 34 (72.7%) abnormality. Other abnormalities were dilacerations 2 (6%), failure of root formation 1 (3.03%), fusion 1 (3.03%) and hypodontia 5 (15.2%).

Supernumerary teeth were found in total of 20 cases. They were mostly located mesial to maxillary canines; 7 (35%) and 5 (25%) supernumerary teeth mesial to right maxillary canine and left maxillary canine respectively. Others 8 (40%) were located distal to central incisors.

**Table 1:** Prevalence of dental anomalies according to gender (n = 208).

Anomalies	Presence/ Absence	Sex	
		Female n (%)	Male n (%)
Agenesis of tooth/teeth	Presence	69 (42.6)	93 (57.4)
	Absence	19 (41.3)	27 (58.7)
Supernumerary teeth	Presence	05 (25.0)	15 (75.0)
	Absence	83 (44.1)	105 (55.9)
Morphological anomalies	Presence	12 (36.4)	21 (63.6)
	Absence	76 (43.4)	99 (56.6)
Positional Anomalies	Presence	16 (29.6)	38 (70.4)
	Absence	72 (46.8)	82 (53.2)
Any kind of dental anomalies	Presence	80 (42.6)	108 (57.4)
	Absence	8 (40.0)	12 (60.0)

**Table 2:** Prevalence of dental anomalies in different types of cleft (n = 208).

Type of cleft	Agenesis n (%)		Supernumerary Teeth n (%)		Morphological Anomalies n (%)		Positional Anomalies n (%)		Any one dental Anomaly n (%)	
	+	-	+	-	+	-	+	-	+	-
Complete	145	39	20	164	30	154	49	135	168	16
(n =184)	(78.8)	(21.2)	(10.9)	(89.1)	(16.3)	(83.7)	(26.6)	(73.4)	(91.3)	(8.7)
Incomplete	16	07	0	23	03	20	05	18	19	04
(n = 23)	(69.6)	(30.4)	(0)	(100)	(13.0)	(87.0)	(21.7)	(78.3)	(82.6)	(17.4)
Laterality Unilateral (n = 151)	114 (75.5)	37 (24.5)	13 (8.6)	138 (91.4)	21 (13.9)	130 (86.1)	41 (27.2)	110 (72.8)	137 (90.7)	14 (9.3)
Bilateral (n = 56)	47 (83.9)	09 (16.1)	07 (12.5)	49 (87.5)	12 (21.4)	44 (78.6)	13 (23.3)	43 (76.8)	50 (89.3)	06 (10.7)

**Table 3:** Frequency of teeth involved in different anomalies.

Type of dental anomalies		Tooth number	Frequency n (%)	Total n (%)
Morphological abnormalities	Dilacerations	22	1 (3.03)	
		21	1 (3.03)	
	Failure of root formation	11	1 (3.03)	
	Fused	24,25	1 (3.03)	
	Hypodontia	12	2 (6.06)	33 (100)
		22	3 (9.09)	
	Peg shape	12	9 (27.27)
		22	15 (45.45)	
Type of ectopia	Impacted	12	1 (7.69)	
		45	1 (7.69)	
		23	1 (7.69)	
		22	2 (15.38)	
		23	1 (7.69)	113 (100)
	Palatally placed	14	4 (30.77)	
	Horizontally placed	11	1 (7.69)	
	Transposition	22,23	1 (7.69)	
		13,14	1 (7.69)	
Type of malalignment	Rotated	21	23 (56.10)	
		11	10 (24.39)	
	Tilted	11	1 (2.44)	
		21	4 (9.76)	
		22	1 (2.44)	
	Upside down	21	2 (4.88)	
				41(100)

## DISCUSSION

In the current study age of the patient ranged from 7 to 27 years with the mean age of 12.5 years and majority of the patients were male. This age group may be due to the fact that most of our patients came for alveolar bone graft and or for orthodontic teeth correction at 7-13 years of age. This gender differences in the prevalence of oral clefts is well supported by the study done by Fogh-Andersen,^6^ 1967 where males are affected more often than females and show more severe clefting.

In this study 184 (88.5%) of the study patients showed complete cleft, 24 (11.1%) showed incomplete cleft with one case of isolated cleft palate. Majority of the patients i.e. 151 (72.7%) had unilateral clefts and only 56 (26.9%) of the study patients had bilateral clefts. Perhaps, the high prevalence of complete clefts compared to incomplete clefts and isolated cleft palate may be due to the fact that panoramic radiographs were done in cleft patients at the time of secondary alveolar bone grafting or for orthodontic treatment.

Presence of at least one dental anomaly in the study population was found to be 188 (90.4%). This frequent occurrence may be attributed to the cleft itself or to the early surgical correction of the defects.^5^ Given that the timing of the primary lip and secondary palate repair: 3-6 and 9-12 months, respectively^7^ coincides with the crown completion of anterior primary teeth and the calcification of upper permanent incisors, surgical manipulation and tissue scarring can affect both stages in primary and permanent anterior teeth. Surgery can also obliterate initiation and calcification of posterior permanent tooth buds or cause displacements and rotations of teeth, possibly explaining the occurrence of agenesis of posterior permanent teeth (i.e., premolars), impactions, and dental malpositions.^8^

The lack of fusion between the mesial nasal and maxillary prominences during the primary palate formation can result in insufficient mesenchyme to support the formation of tooth buds. Alternatively, the cleft can result in an extension of dental lamina, which can develop into extra teeth or can cause division of the tooth buds, resulting in supernumerary teeth. If the remaining tooth bud's tissue is defective or incapable to develop into a viable tooth, microdontia or agenesis could occur (Ranta 1986).^8^

Tooth agenesis is the most clearly recognized dental abnormality in humans. The frequency of tooth agenesis(both in and outside the cleft region) is significantly high in persons with clefts compared with the general population.^9^ Our study agrees with this fact by showing 161 (77.9%) prevalence of dental agenesis in cleft population. While a cross-sectional descriptive study done in 601 orthodontic patients at Tribhuvan University Teaching Hospital and Dental Villa-Orthodontic Center and Speciality Dental Clinic, Kathmandu, Nepal in 2019 showed the prevalence of dental agenesis in general population to be 7.48% excluding the third molar.^10^

This high prevalence of dental agenesis among dental anomalies in cleft population is well supported by scientific literature. Al Jamal et al showed 66.7% of CL/P sample had missing teeth in Jordanian population.^5^ In a study by Al Kharboush et al, the most common dental anomaly was hypodontia, which occurred in 123 (66.8%) subjects of Saudi cleft lip and palate patients.^3^ Similarly, Reina Colombo et al^11^ showed 93% dental agenesis in cleft patients of Colombo and Ribeiro Paranaiba et al^12^ showed 47.5% dental agenesis in Brazilian population which was the most commonest anomalies among the cleft lip and palate patients in their respective studies. The percentage of dental agenesis varies largely in these studies including our study.

Positional anomalies like ectopic eruption, impaction, transposition, rotation and tilted teeth were seen among 54 (26%) patients in this study. Positional anomalies was seen in higher proportion among the unilateral cleft patients than bilateral clefts patients while other dental anomalies like agenesis, presence of supernumerary teeth and morphological anomalies were found more in bilateral clefts compared to unilateral cleft patients. Previous literature shows varied prevalence of positional anomalies such as 30.8% ^5^, 12.3% ^3^ and 22.1%^11^. In our study maxillary central incisors were the most common teeth to show malalignment (rotation, tilted or upside down) showing higher prevalence on left side than right side. However, there was almost uniform distribution of teeth with ectopic eruption in maxillary dentition. The various types of ectopia observed in the study were: impaction, palatal orientation, horizontal orientation and transposition of teeth.

The supernumerary teeth were seen among 20 (10%) of the patients and microdontia including peg shaped laterals were seen among 33 (15.9%) of the patients in this current study. The prevalence of supernumerary teeth in this study is well supported by studies like Al-Kharboush who indicated 12.5% prevalence of supernumerary teeth^3^, Al Jamal et al^5^ mentioned 16.7% had supernumerary teeth in their study and Tereza who indicated 12% prevalence of supernumerary teeth.^13^ However the prevalence of microdontia in our study was much less than studies done by Al Jamal (37%) and Al Kharboush (47.5%).

Previous reports have established that oral clefts present a sexual dimorphism: CL/P is more common in males and severe forms are more common in males.^14^ This study agrees with this statement, as we have more male patients and showed higher proportion of dental anomalies compared to females. We also observed differences in associations of dental anomalies with cleft type. All dental anomalies were seen in higher proportions among patients with complete clefts compared to patients with incomplete clefts.

The absence of fusion between the maxillary and medial nasal processes that resulted in the CL/P may be a contributing factor for the various anomalies that affect the lateral incisor. This could explain the frequent absence of lateral incisors or their distal or mesial location with respect to the cleft, as well as the presence of supernumerary teeth in the same region.^5^ Presence of supernumerary teeth in this region is because the tooth buds of the permanent lateral incisors are susceptible to modification, or division, possibly being divided by the cleft. Presence of supernumerary teeth in this region is because the tooth buds of the permanent lateral incisors are susceptible to modification, or division, possibly being divided by the cleft.^3^

Published studies on dental anomalies in subjects with CLP have shown that the maxillary permanent lateral incisors are the most susceptible tooth to be affected in the vicinity of the cleft.^15^ This fact was well supported by our study. Maxillary laterals were the most common teeth to be missing in our cleft populations with higher prevalence on left side compared to right side. Among morphological anomalies, the most common abnormality was peg shaped maxillary lateral incisors 34 (72.72%). Maxillary left lateral incisors 22 (45.45%) were more affected than their right counterpart 12 (27.27%). The supernumerary teeth were all present at the cleft site either mesial to canines or distal to central or lateral incisors, thus supporting the literatures regarding the location of supernumerary teeth in CL/P.^5^

Our study represents a thorough and complete description of dental anomalies present in a sample of cleft lip and palate patients, however a much larger multi-centre sample is perhaps required to determine the relationship of each dental anomaly with cleft type and laterality of cleft.

## CONCLUSIONS

Dental anomalies are highly prevalent among patients with CL/P which was observed in higher proportion among males compared to females in this study. The most common anomaly was found to be dental agenesis. Peg shaped laterals and hypodontia were the common morphological abnormalities presented by lateral incisors when present. Other anomalies like ectopic eruption were also more depicted by the teeth adjacent to the cleft site and supernumerary teeth whenever present were present at the cleft site.

## Conflict of Interest

**None.**
